# Neighborhood Urban Environmental Quality Conditions Are Likely to Drive Malaria and Diarrhea Mortality in Accra, Ghana

**DOI:** 10.1155/2011/484010

**Published:** 2011-06-21

**Authors:** Julius N. Fobil, Alexander Kraemer, Christian G. Meyer, Juergen May

**Affiliations:** ^1^Infectious Disease Epidemiology Group, Bernhard Nocht Institute for Tropical Medicine, Bernhard Nocht Str. 74, 20359 Hamburg, Germany; ^2^Department of Public Health Medicine, School of Public Health, University of Bielefeld, P.O. Box 100131, 33501 Bielefeld, Germany; ^3^Department of Biological, Environmental & Occupational Health Sciences, School of Public Health, University of Ghana, P.O. Box LG13, Legon, Ghana; ^4^Department Molecular Medicine, Bernhard Nocht Institute for Tropical Medicine, Bernhard Nocht Str. 74, 20359 Hamburg, Germany

## Abstract

*Background*. Urbanization is a process which alters the structure and function of urban environments. The alteration in the quality of urban environmental conditions has significant implications for health. This applies both to the ecology of insect vectors that may transmit diseases and the burden of disease. *Study Objectives*. To investigate the relationship between malaria and infectious diarrhea mortality and spatially varied neighborhood environmental quality conditions in a low-income economy. *Design*. A one time point spatial analysis of cluster-level environmental conditions and mortality data using principal component analysis (PCA), one-way analysis of variance (ANOVA) and generalized linear models (GLMs). *Methods*. Environmental variables were extracted from the Ghana Census 2000 database while mortality data were obtained from the Ghana Births and Deaths Registry in Accra over the period 1998–2002. *Results*. Whereas there was a strong evidence of a difference in relative mortality of malaria across urban environmental zones of differing neighborhood environmental conditions, no such evidence of mortality differentials was observed for diarrhea. In addition, whereas bivariate analyses showed a weak to strong evidence of association between the environmental variables and malaria mortality, no evidence of association was found between diarrhea mortality and environmental variables. *Conclusion*. We conclude that environmental management initiatives intended for infectious disease control might substantially reduce the risk of urban malaria mortality and to a less extent that for urban diarrhea mortality in rapidly urbanizing areas in a low-income setting.

## 1. Background

**Figure 1:**
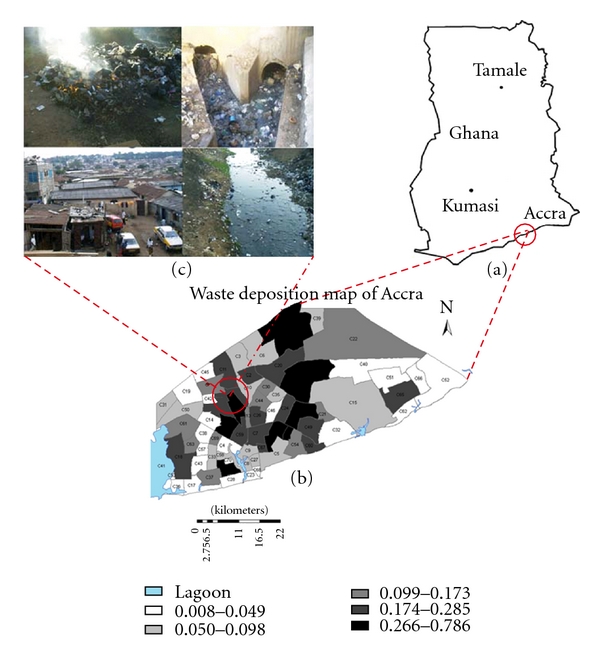
Map of the study area, Accra, Ghana. (a) is the map of Ghana showing the geographic position of Accra. (b) Shows the 70 census clusters which were used as the units of analysis. (c) Shows the amplification of the neighbourhood environmental conditions (i.e., uncontrolled solid waste incineration, choked open drain, stagnant water in an open drain, and dilapidated housing conditions) as pertain in the census clusters.

Life within urban spaces has become a characteristic feature of modern human ecology as the global number of cities has increased exponentially and expanded rapidly over the past two centuries following industrialization and sustained economic growth [1–4]. While cities are seen as sources of creativity and technology, often conceived as the engines for economic growth, they are also in many cases; especially in low-income economies centers, of pervasive poverty, economic inequality, and health hazards from harmful environmental agents [1, 3, 4]. Rapid urbanization has been widely reported to alter the structure of urban subsystems, a process which has considerable implication for health [1, 3–7]. Ample evidence exists to show that uncontrolled and unplanned expansion, including sheer increase in urban population numbers, combine to accelerate the deterioration of urban environmental quality conditions such as waste deposition, outdoor air pollution, and water pollution [1, 3, 8–12].  Figure 1 shows a typical scenario in an urban slum in Accra, Ghana, displaying distinct environmental quality conditions. 

The mere lack of sanitation, poor infrastructure, and deplorable urban living conditions are believed to increase the risk of infectious diarrhea [1–3, 9–11, 13]. The effectiveness of a city-wide sanitation intervention on diarrhea in urban centers in low-income countries has recently been demonstrated [2, 10, 13]. However, the influence of neighborhood environmental conditions on infectious diarrhea mortality is not completely understood [5, 14–17]. In the case of malaria, the most important vector-borne disease, it not only still remains, despite deployment of better control strategies, a leading cause of morbidity and mortality, but it also exacerbates socioeconomic conditions, particularly in poor communities [18–22]. Unprecedented urbanization, along with declining environmental conditions, might have profound implications for the epidemiology and control of malaria, as the relative disease burden increases among urban dwellers [3, 4, 6, 7]. A recent review and modeling study demonstrated that entomologic inoculation rates (EIR) in cities in urbanizing areas were rising and ranged up to 54 per year, depending on the degree of urbanization, the spatial location within a city, and the overall living conditions [10]. Although the epidemiology of urban malaria is now fairly understood, there is still wide disagreement about its transmission dynamics [10, 18, 19, 21–23]. While it is generally accepted that rapid urbanization creates environmental conditions that favor insect vector breeding, others argue that urban pollution tends to inhibit the growth and development of mosquito larvae [10, 18, 21, 22]. Understanding how the risk to human health may change as a result of variations in environmental conditions will be a key priority in the context of climate change and urban health, especially in lower-income countries with fragile structures that are most sensitive to climate events and extreme weather conditions [3, 24–28]. 

Therefore, the main goal of this study was not only to investigate the relationship between spatially varied neighborhood urban environmental quality and malaria and diarrhea mortality, but also to find out if the fraction of deaths due to malaria and diarrhea differed across environmental zones in an urbanizing area in a low-income economy. 

## 2. Methodology and Analytic Approach

The data on neighbourhood urban environmental quality conditions were obtained from the 2000 Census database while health data, totalling 24,716 deaths and coded according to ICD-9, were obtained from the Ghanaian Vital Registration System (VRS) that routinely reported all-cause deaths for years 1998 to 2002. Different data handling procedures were applied to the two datasets because the measures of mortality and environmental quality differed significantly. 

In this analysis, four broad categories of environmental variables were obtained from the census database, namely (1) “population and waste generation”, (2) “water supply and sanitation”, (3) “hygiene conditions”, and (4) “built structure, form, construction material type and living arrangements”. In all, a total of 65 single environmental variables were extracted from the census database under the four distinct subcategories together and summary measures (e.g., the proportions of cluster-level conditions) appropriately computed. In order to find out whether differences in malaria and diarrhea mortalities existed across different environmental zones, we first explored the large number of environmental variables under each subcategory for determination of the direction of their eigenvectors using principal component analysis (PCA). In a data reduction strategy, we again used PCA to decompose the variables under each subcategory into a manageable unidirectional variable which was employed to develop an ordinal scale of urban environmental *zonations* at differing levels of environmental quality conditions [5]. The resulting environmental zones were nominally defined as “extremely deteriorated zone”, “moderately deteriorated zone”, and “least deteriorated zone” representing worst, somewhat worse, and good environmental conditions, respectively. We then compared the differences in malaria and diarrhea mortality levels in the different environmental zones using a one-way analysis of variance (ANOVA). In a subsequent step, we assessed the linear association between the environmental variables on the one hand and malaria and diarrhea mortalities on the other hand using generalized linear models (GLMs). In order to deal with multicollinearity in the linear association analysis, we conducted correlations using (STATA version 9, College Station, Tex, USA) and examined the correlation matrix as well as the variance inflation factor (VIF) of the exploratory variables. Variables with VIF greater than 4 were either modified or excluded completely from the analysis if modification did not make sense. Overall, a total of 60 variables were maintained for use in the ANOVA and linear regression analyses after dealing with multicollinearity.

For the health data, we allocated all death records to the 70 census clusters for which urban environmental variables were available according to the place of residence prior to death and computed surrogate proportional mortality ratios (PMRs) as fractions of cluster-level deaths due to malaria and diarrhoea. Overall, 24,716 all-cause deaths events were recorded for urban Accra for the period from 1998 to 2002, and out of which 1,292 and 1,001 were deaths attributed to malaria and diarrhea, respectively. The standard definition of a PMR is the observed proportion of deaths due to specific-cause mortality in an exposed population, divided by the expected proportion of deaths due to the specific cause (i.e., the expected proportion being the number of deaths due to the specific cause over all-cause mortality in a standard or reference population). To obtain cluster-level fraction of deaths due to malaria or diarrhea, we worked backwards, multiplying cluster level PMRs by the expected proportion, that is, the proportion of malaria or diarrhea in a reference population. We also computed relative mortality (RM), that is, the relative risk of mortality in the different zones as a ratio of the mean of the fraction of deaths due to malaria/diarrhea in each environmental zone to the mean of the fraction of deaths due to malaria/diarrhea in the least deteriorated zone. The effects of the neighbourhood urban environmental quality conditions on mortality were assessed by comparing the means of the fraction of deaths due to malaria and diarrhea whereas the relative mortalities across the different environmental zones, and the association between environmental quality and malaria and diarrhea mortality were investigated using bivariate and multiple linear regression analyses. 

## 3. Results

In an analysis to assess the influence of neighbourhood urban environmental quality on the fraction of deaths due to malaria and diarrhea, we present the means of the fraction of deaths (mean fraction) and their respective relative mortalities (RMs) in scenarios of differing urban environmental quality conditions in a rapidly urbanizing area in a low-income economy (Tables 1 and 2). 

Table 1 compares the fraction of cluster-level deaths due to malaria across environmental zones defined on the basis of four subcomponents of environmental quality, namely, (a) total waste generated in relation to population (per capita generation), (b) water supply and sanitation, (c) hygiene facilities/conditions, and (d) housing structure, form, and construction material type. On the basis of the “population and waste generation” sub-component, there was strong evidence of a difference (*P* = .018) in the risk of urban malaria mortality between the least deteriorated zone (mean fraction = 0.029, 95% CI: 0.011–0.045) and the moderately deteriorated zone (mean fraction = 0.051, 95% CI: 0.04–0.059) with a relative mortality [RM] = 1.77 between the two zones. However, there was no evidence of a difference (*P* = .061) in the risk of malaria mortality between the reference zone (least deteriorated zone) and extremely deteriorated (mean fraction = 0.50, 95% CI: 0.031–0.070) zone. Regarding “water supply and sanitation”, a strong evidence of a difference in the risk of malaria mortality was observed both between the least deteriorated zone (*P* = .004; mean fraction = 0.024; 95% CI: 0.012–0.036) and the moderately deteriorated zone (mean fraction = 0.049, 95% CI: 0.040–0.058) on the one hand and between the extremely deteriorated zone (mean fraction = 0.054, *P* = .007, 95% CI: 0.035–0.072) and the least deteriorated zone on the other hand. The relative risks of death due to malaria in the moderately deteriorated (RM = 2.041) and extremely deteriorated (RM = 2.221) zones were approximately the same. Whereas a strong evidence of a difference (*P* = .012) in malaria mortality was observed between the extremely deteriorated (mean fraction = 0.024, 95% CI: 0.011–0.038) and the reference zones for “hygiene facilities/conditions”, no evidence of a difference (*P* = .690) was observed between the moderately deteriorated and the reference zones (mean fraction = 0.052, 95% CI: 0.040–0.062). The relative risk of death due to malaria in the zone of moderately deteriorated hygiene conditions (RM = 0.931) and in the zone of extremely deteriorated hygiene conditions (RM = 0.470) was remarkably different although the fraction of deaths due to malaria in the two zones was lower than that in the least deteriorated hygiene conditions. The “housing structure and form, construction materials, and living arrangement” component of urban environment was classified into three categories, namely, (1) “extreme-slum”, (2) “moderate-slum” and (3) “non-slum” conditions. While we observed a strong evidence of malaria mortality (*P* = .004) between the zone of moderate slum conditions (mean fraction = 0.050, 95% CI: 0.040–0.061) and the zone of least slum conditions (mean fraction = 0.028, 95% CI: 0.019–0.037), no evidence of mortality difference (*P* = .112) was observed between the reference slum conditions and the zone of moderate slum conditions (Table 1).

Table 2 summarizes urban diarrhea mortality burden in the different zones. In contrast to the mortality pattern observed for malaria, the evidence of diarrhea mortality difference across the different spatially distinct environmental zones was not as striking. While there was moderate evidence of diarrhea mortality difference (*P* = .035) across the 3 slum grades, there was no evidence of diarrhea mortality differential in the other components of environmental quality except for a moderate evidence of difference (*P* = .036) between moderately deteriorated (mean fraction = 0.035, 95% CI: 0.029–0.042) and least deteriorated zones with respect to hygiene conditions.

As the analyses not only assessed whether or not there were differences in the urban mortalities across the different environmental zones, we evaluated the relationship between malaria- and diarrhea-specific mortalities on the one hand and the neighbourhood environmental quality conditions on the other as an additional interest using bivariate regression analyses. 

**Figure 2:**
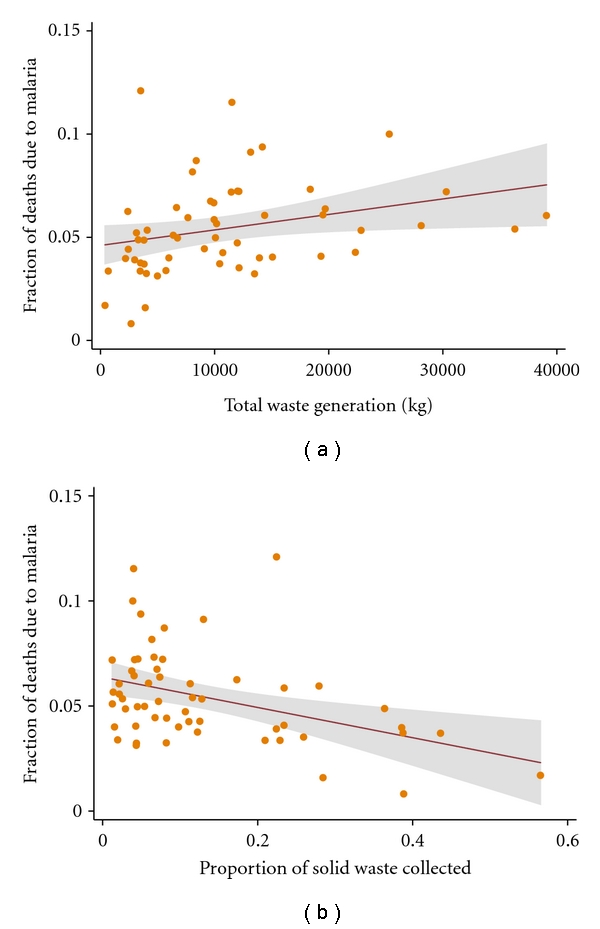
The nature of the bivariate associations between malaria mortality and solid waste generation. The points represent the fraction of deaths due to malaria and the metric of solid wastes. The solid lines represent the regression line of best fit, and the grey area represents their confident intervals (CIs). (a) Shows the total amount of solid waste generated as a function of the fraction of cluster-level deaths due to malaria. (b) Shows wastes collection rate as a function of the fraction of cluster-level deaths due to malaria.

In an assessment of the relationship between solid waste generation and malaria mortality, a unit increase in solid generation resulted in an increase in malaria mortality (Figure 2(a)). While total solid waste generated accounted for approximately 10 percent of the observed variation in malaria mortality (*R* ^2^ = 0.11), we in addition observed a strong evidence of association (*P* = .005, coefficient = 1.16×10^−06^, 95% CI: 3.6 × 10^−07^–2 × 10^−06^) between the two variables. On the contrary, the relationship between malaria mortality and per capita waste generation showed only weak evidence of association (*P* = .050, coefficient = 0.061, 95% CI: 1.0 × 10^−4^–0.123).  Figure 2(b) illustrates the relationship between solid waste collection rate, that is, the proportion of the solid wastes collected out of the total amount generated and malaria mortality. A unit increase in waste collection rate resulted in a unit decrease in malaria mortality. There was strong evidence of association (*P* = .002, coefficient = −0.075, 95% CI: −0.120–−0.029) between waste collection rate and the fraction of cluster-level deaths that were attributed to malaria. The amount of variation in urban malaria mortality explained by the variation in waste collection rate was 14 percent (*R* ^2^ = 0.1370).

Similarly, an increase in the proportion of households connected to the central sewer system led to a decrease in malaria mortality. The amount of variation in malaria mortality that was explained by the variation in the proportion of households connected to the central sewer system was 15 percent (*R* ^2^ = 0.1465). A strong association (*P* = .001, coefficient = −0.083, 95% CI: −0.1311–−0.0344) was observed between the proportion of households connected to the sewer system and malaria mortality.

**Figure 3:**
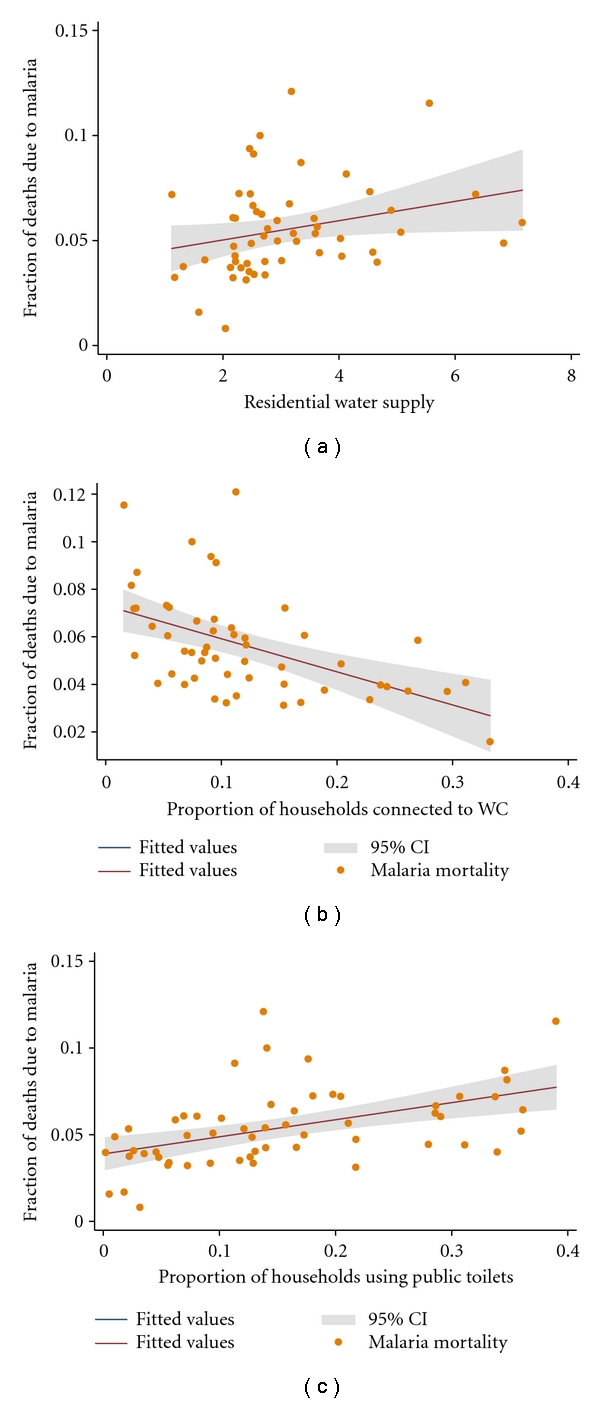
The nature of the bivariate associations between malaria mortality and urban water supply and sanitation. The points represent the fraction of deaths due to malaria and the metric of urban water supply and sanitation. The solid lines represent the regression line of best fit, and the grey area represents their confident intervals (CIs). (a) Shows the number of residential water supply sources as a function of the fraction of cluster-level deaths due to malaria. (b) Shows the proportion of households with WCs as a function of the fraction of cluster-level deaths due to malaria. (c) Shows the proportion of households using public toilets as a function of the fraction of cluster-level deaths due to malaria.

Figure 3(a) shows the association between number of residential water supply sources per neighbourhood and malaria mortality, while Figure 3(b) depicts the relationship between the proportion of households connected to the water closet (WC) and malaria mortality. Although malaria mortality was strongly associated with both the number of residential water supply sources per household (*P* = .008, coefficient = 0.007, 95% CI: 0.002–0.013) and the proportion of households connected to WC (*P* < .001, coefficient  = −0.087, 95% CI: −0.129–−0.044), the amount of evidence of association for the proportion of household connected to WC was stronger compared to solid waste collection rate. An increase in the proportion of households connected to the WC resulted in a decrease in malaria mortality, and the amount of variation in the mortality explained by the variation in the proportion of households connected to the WC was approximately 20 percent (*R* ^2^ = 0.1966).  Figure 3(c) gives the relationship between the proportion of households using public toilets and malaria mortality. Although the use of public toilets has not been previously reported to influence malaria transmission, it was observed that an increase in the proportion of households using public toilets resulted in an increase in malaria mortality. The amount of variation in malaria mortality explained by a unit increase in the proportion of households depending on public toilets as hygiene facilities was approximately 15 percent (*R* ^2^ = 0.1498). Strong evidence of association was observed between the proportion of urban households using public toilets and malaria mortality (*P* = .001, coefficient  = 0.099, 95% CI: 0.042–0.157).

We further assessed the influence of hygiene facilities such as shared bath facility, own bath facility, public bath, and shared cubicle, whether or not hygiene facility was inside the household in malaria mortality. An assessment of the relationship between the proportion of households without hygiene facilities was observed to show a positive association with malaria mortality. The amount of variation in urban malaria mortality explained by the proportion of households without hygiene facilities was roughly 8 percent (*R* ^2^ = 0.0817), with a strong evidence of association (*P* = .016, coefficient = −0.193, 95% CI: −0.350–−0.037) between the variables. In both bivariate and multi-variate analyses, the response of malaria mortality to the different hygiene variables varied widely in direction, strength, degree, and levels of association. For example, while a unit increase in the proportion of households with own-bath facilities and those with shared-bath facilities resulted in a decrease in malaria mortality, the degree of variability explained was approximately 13 percent (*R* ^2^ = 0.1249) for households with own-bath facilities and 7 percent (*R* ^2^ = 0.0701) for households with shared-bath facilities. Moreover, while the strength of the evidence of association between malaria mortality and the proportion of households with own-bath facilities was very strong (*P* = .003, coefficient = −0.049, 95% CI: −0.081–−0.018), only moderate evidence of association (*P* = .027, coefficient = −0.070, 95% CI: −0.133–−0.008) was observed between urban malaria mortality and the proportion of households with shared-bath facilities.

**Figure 4:**
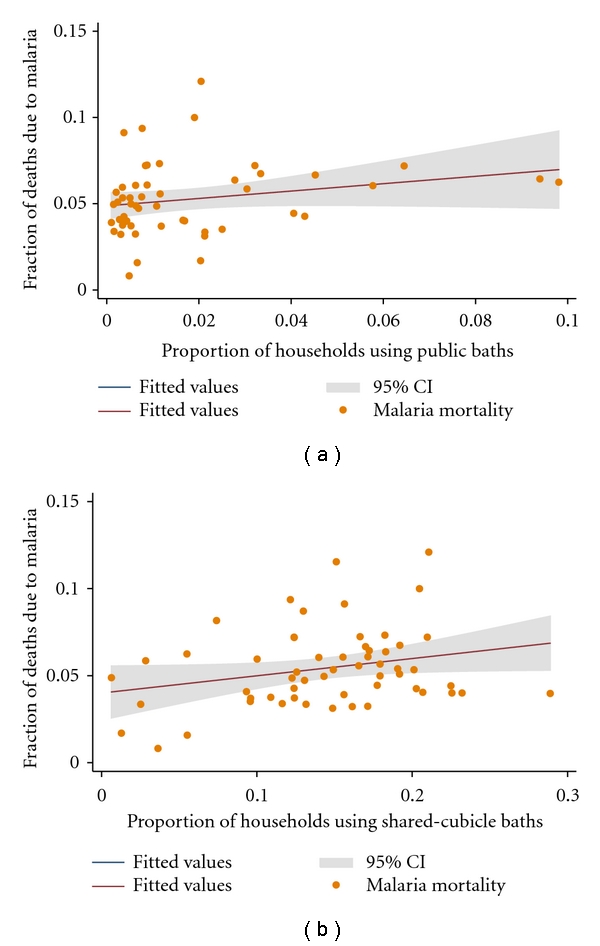
The nature of the bivariate associations between malaria mortality and neighbourhood hygiene conditions. The points represent the fraction of deaths due to malaria and the metric of solid wastes. The solid lines represent the regression line of best fit, and the grey area represents their confident intervals (CIs). (a) Shows the proportion of households using public baths as a function of the fraction of cluster level deaths due to malaria. (b) Shows the proportion of households using shared-cubicle baths as a function of the fraction of cluster level deaths due to malaria.

Figure 4 compares the differences in the levels and strengths of association between malaria mortality and the proportion of households using public bath facilities on the one hand and the association between the proportion of households using shared-cubicle baths and malaria mortality on the other hand.

In both instances, a unit increase in the explanatory variable (i.e., the proportion of households using public baths: Figure 4(a) and the proportion of households using shared cubicle-baths: Figure 4(b)) resulted in a corresponding increase in malaria mortality. However, while the amount of variation in malaria mortality explained by the proportion of households using public bath facilities was 8 percent (*R* ^2^ = 0.0817), the amount of variability in malaria mortality explained by the proportion of households using shared-cubicle baths was 12 percent (  *R* ^2^ = 0.1200). There was a strong evidence of association (*P* = .003, coefficient = 0.163, 95% CI: 0.056–0.269) between malaria mortality and the proportion of households using shared-cubicle baths. Furthermore, although the association between malaria mortality and the proportion of households using public baths was strong (*P* = .016, coefficient  = 0.173, 95% CI: 0.033–0.314), it was slightly weaker than that between malaria mortality and the proportion of households using shared-cubicle baths (Figure 4(b)).

Another aspect of neighbourhood environmental quality that was assessed to determine its influence on malaria mortality was housing structure, housing characteristics, and living arrangements. The city of Accra is poorly planned, composed largely of light-density buildings in a sprawling landscape. The form and structure of built-up areas represent a diversity of environmental archetypes of different outlook and varying levels of neighbourhood environmental quality conditions. It is not very clear how the different building and structure types influence infectious disease transmission and in particular, malaria and diarrhea mortalities in rapidly urbanizing areas in a low-income economy. In this analysis; therefore, our interest did not only focus on how the specific neighbourhood environmental quality condition influenced infectious disease mortalities, but also how the different structural designs and types were linked to urban mortalities. Classified broadly under the heading “housing construction material type and living arrangements”, both bivariate and multiple linear regression procedures were conducted on several variables related to the structure and form of buildings, construction material type, living arrangements, that is, number of persons per unit, and so forth. Under this component of the physical environment, of a total of 15 variables (e.g., proportion of standalone structures, proportion of semidetached structures, proportion, and flats/apartment structures, proportion of compound structures, proportion of huts, proportion of mud-brick structures, proportion concrete-brick structures, proportion of bamboo structures, etc.) considered for both bivariate and multiple regression analyses, only 3 showed significant association with urban malaria mortality. The 3 variables were: (1) the proportion of standalone structures, (2) the proportion of flats/apartment buildings and (3) the proportion of compound structures which housed multiple households. 

**Figure 5:**
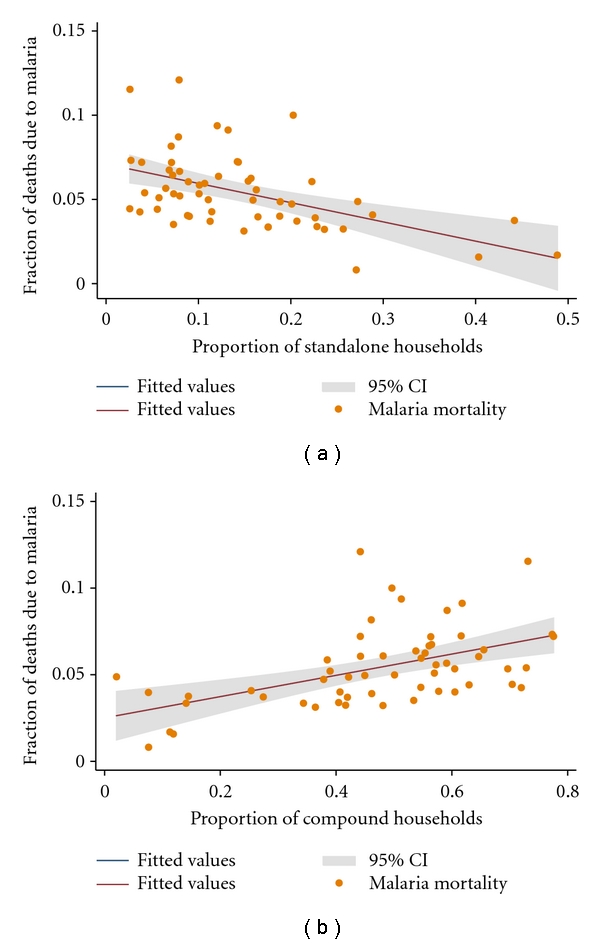
The nature of the bivariate associations between malaria mortality and housing and living arrangements. The points represent the fraction of deaths due to malaria and the metric of solid wastes. The solid lines represent the regression line of best fit, and the grey area represents their confident intervals (CIs). (a) Shows the proportion of standalone households as a function of the fraction of cluster level deaths due to malaria. (b) Shows the proportion of compound households as a function of the fraction of cluster level deaths due to malaria.

Figure 5(a) displays the relation between the proportion of standalone structures and urban malaria mortality. Here, it was observed that a unit increase in the proportion of standalone structures led to a large decline in urban malaria mortality with the amount of variation in urban mortality that was explained by a unit increase in the proportion of standalone structures being as large as 21 percent (*R* ^2^ = 0.2051). There was a strong evidence of association between the proportion of standalone building structures and urban malaria mortality (*P* < .001, coefficient  = −0.118, 95% CI: −0.174–−0.062).

In a penultimate assessment, a unit increase in the proportion of flats/apartment structures resulted in a decrease in malaria mortality. Approximately 7 percent (*R* ^2^ = 0.0730) of the variation in malaria mortality was explained by a unit increase in the proportion of flats/apartment building structures. Nonetheless, there was a moderate evidence of association (*P* = .024, coefficient = −0.098, 95% CI: −0.183–−0.014) between the proportion of flats/apartment structures and malaria mortality compared to the strong evidence of association observed between malaria mortality and the proportion of standalone building structures.

In a more or less final assessment, the relationship between the proportion of compound structures and urban malaria mortality was evaluated.  Figure 5(b) shows the relationship between the proportion of compound building structures and urban malaria mortality. A unit increase in the proportion of compound structures resulted in an increase in malaria mortality. Twenty-one percent (*R* ^2^ = 0.2126) of the variation in malaria mortality was explained by a unit increase in the proportion of compound building structures. The observed evidence of association between the proportion of compound building structures and malaria mortality was very strong (*P* < .001, coefficient = 0.071, 95% CI: 0.038 0.105). 

## 4. Discussions

Considerable evidence which demonstrates the effects of the physical environment on human health exists [5, 8, 10, 29]. However, no consensus exists on the standard definition of environmental quality classifications that allows for across-study comparisons. In literature, different studies have different environmental classifications, thus, making comparison of study outcomes between and among different studies hard to undertake. 

Both solid and liquid wastes are known to constitute conducive breeding media for insect vectors including the opportunity to create stagnant pools of water for mosquito larval growth. As a consequence, increased waste collection decreases habitat space and capacity for the larvae of *Anopheles* mosquitoes, which are the vectors of malaria parasites. This might lead to a decrease in malaria transmission and, therefore, a reduction in its mortality. In addition, an increase in residential water supply sources would potentially lead to expansion of residential habitat space for mosquito larval growth and survival and was likely the reason for the observed positive association between urban malaria mortality and the number of residential water supply sources. Furthermore, nonremoval of liquid waste may create poodles and standing water conditions favoring breeding of mosquitoes in open drain systems. As a consequence, residential areas with a low sewer connection rate may be noted for high mosquito breeding and thus, be associated with increased malaria transmission. This was probably the explanation for the observed reduction in urban malaria mortality with an increase in the proportion of households that were connected to the sewer system. Our observation are consistent with the findings reported in a number of previous studies on the association between mosquito vector breeding and waste material deposition [7, 21, 30–32]. Like both solid waste collection rate and the proportion of households connected to the sewer system, the higher the proportion of households connected to the WC, the smaller the chance of liquid wastes left to collect as standing pools of water. This has a potential to reduce the size of habitats for mosquito breeding and thus, lowering malaria transmission and perhaps accounted for the decrease in malaria mortality associated with the increase in the proportion of households connected to the WC. 

The observed lower malaria mortality rate in the moderately deteriorated zone compared to the least deteriorated zone for hygiene facilities was a striking anomaly which was probably due to behavioral and lifestyle factors rather than environmental factors. Lifestyle and behavioral factors can upset an otherwise uniform natural association between a health outcome and environmental conditions. For instance, strict observance of malaria control measures by residents in the moderately deteriorated environmental zone could account for the observed difference.

Although several studies have reported increased malaria transmission with deteriorating environmental quality [8, 18, 27, 33–37], no reports of such a relationship between environmental conditions and malaria mortality have been documented. In a study to investigate the risk factors for malaria, children living in houses with mud roofs had significantly higher risk of acquiring malaria compared to those living in iron-sheet-roofed houses (OR 2.6, 95% CI: 1.4–4.7) [22]. 

The lack of evidence of association between the fraction of cluster-level deaths due to diarrhea and environmental conditions was likely a confirmation that the urban environmental initiatives for the control of diarrhoeal diseases (CDD) have been effective and the observed diarrhoeal deaths were not related to the environmental conditions. It is well known that there is a strong association of diarrhea morbidity and areas with poor hygiene and sanitation [2, 9, 10, 13, 16]. In a study to explore how a citywide sanitation intervention altered the magnitude of the relative and attributable risks of diarrhea determinants and the pathways by which those factors affected diarrhea risk, the authors observed that the intervention reduced diarrhea and also changed attributable and relative risks of diarrhea determinants by altering the pathways of mediation [10]. In addition, socioeconomic status was a major distal diarrhea determinant with an attributable risk of 24%. Whereas 90% of risk was mediated by other factors, mostly by poor infrastructure (53%) and lack of sanitation which accounted for 13% of the risk, the remaining 42% mediated by other factors including 18% by lack of sanitation and poor infrastructure [10]. This study observed very weak to very strong associations between neighbourhood urban environmental quality conditions and urban malaria/diarrhea mortalities. As expected, although the study could not establish causal relationships, several instances of strong associations were observed among the environmental, socioeconomic, and health variables. 

## 5. Conclusion

We conclude that the numerous instances of strong associations between the urban environmental variables and malaria mortality are indications that malaria control programs with a strong focus on urban environmental management initiatives would probably lower urban mortalities attributable to malaria in rapidly urbanization areas in resource-poor settings. However, we recommend that the present study be repeated in a longitudinal study design using the Ghana Census 2010 data in order to confirm these associations and to evaluate how much of malaria deaths are attributed to deterioration in urban environmental conditions over time. In addition, we recommend that case-control studies at individual levels be conducted on the variables that were observed to exhibit strong associations in order to establish the causal chain of events linking, socioeconomic conditions, neighbourhood urban environmental quality conditions, and infectious disease mortality. 

## 6. Limitations of the study

A key limitation in ecological designs is the chance of ecological fallacy, but with the large mortality data available there was no any other design possible. In addition, the PMR_fd_s were computed using all-cause mortality as the denominator, and so if the risk of mortality for one of the components of all-cause mortality varied by cluster, then this could affect the summary/outcome measure PMR_fd_ and, therefore, bias the final results of analysis. A way to deal with this problem will be to exclude such components, but unfortunately, we did not have the liberty of telling *apriori* if any of the components of all-cause mortality exhibited differential mortality risk by cluster and, therefore, this bias could not be controlled for. Another way to deal with this bias will be to use a specific cause of death known to have a uniform risk level across all clusters as the denominator for both the observed proportion and the expected proportion. But, then again, this also requires knowledge of risk distribution by cause in the study area and which unfortunately was not available for the study area. 

## Figures and Tables

**Table 1:** Malaria mortality in different urban environmental zones.

Urban environmental variable	Zonation	RM	Mean fraction	*P*-value	95% CI
Total waste generation in relation to cluster population	Extremely deteriorated	1.755	0.050	.061	0.031 0.070
	Moderately deteriorated	1.769	0.051	.018	0.041 0.059
	Least deteriorated	1.000	0.029	—	0.011 0.045

Water supply and sanitation facilities	Extremely deteriorated	2.220	0.054	.007	0.035 0.072
	Moderately deteriorated	2.041	0.049	.004	0.040 0.058
	Least deteriorated	1.000	0.024	—	0.012 0.036

Hygiene facilities	Extremely deteriorated	0.470	0.024	.012	0.011 0.038
	Moderately deteriorated	0.931	0.048	0.690	0.039 0.058
	Least deteriorated	1.000	0.052	—	0.040 0.062

Housing structure & form, construction material type, and arrangement	Extreme slum	2.349	0.066	.004	0.052 0.080
	Moderate slum	1.794	0.050	.112	0.040 0.061
	Non slum (Well built)	1.000	0.028	—	0.019 0.037

Zonations: represent the different environmental ecotypes with discretely distinct environmental quality conditions. RM: represents relative mortality computed as the ratio of the mean of the fraction of cluster-level deaths due to a specific cause to that of the baseline situation, that is, the fraction of cluster-level deaths due to the specific cause in the least deteriorated zone. Mean fraction: represents the mean of the fraction of cluster-level deaths due to a specific cause. 95% CI: represents 95-percent confidence intervals of the means.

**Table 2:** Diarrhea mortality in different urban environmental zones.

Urban environmental variable	Zonation	RM	Mean fraction	*P*-value	95% CI
Population, water, housing, and waste generation	Extremely deteriorated	1.258	0.036	.411	0.024 0.049
	Moderately deteriorated	1.728	0.050	.160	0.033 0.066
	Least deteriorated	1.000	0.029	—	0.016 0.036

Water supply and sanitation facilities	Extremely deteriorated	1.010	0.040	.986	0.029 0.052
	Moderately deteriorated	1.100	0.044	.799	0.028 0.060
	Least deteriorated	1.000	0.040	—	0.013 0.067

Hygiene facilities	Extremely deteriorated	1.740	0.067	.036	0.016 0.119
	Moderately deteriorated	0.917	0.035	.618	0.029 0.042
	Least deteriorated	1.000	0.039	—	0.029 0.048

Housing construction material type and arrangement	Extreme slum	1.349	0.060	.035	0.027 0.094
	Moderate slum	0.700	0.031	.035	0.024 0.039
	Least slum (Well built)	1.000	0.045	—	0.038 0.052

Zonations: represent the different environmental ecotypes with discretely distinct environmental quality conditions. RM: represents relative mortality computed as the ratio of the mean of the fraction of cluster-level deaths due to a specific cause to that of the baseline situation that is the fraction of cluster-level deaths due to the specific cause in the least deteriorated zone. Mean fraction: represents the mean of the fraction of cluster-level deaths due to a specific cause. 95% CI: represents 95-percent confidence intervals of the means.
